# Outcomes following lymphaticovenous anastomosis (LVA) for 100 cases of lymphedema: results over 24-months follow-up

**DOI:** 10.1007/s10549-020-05839-4

**Published:** 2020-08-07

**Authors:** Shan S. Qiu, Tim Pruimboom, Anouk J. M. Cornelissen, Rutger M. Schols, Sander M. J. van Kuijk, René R. W. J. van der Hulst

**Affiliations:** 1 Department of Plastic and Reconstructive Surgery, Maastricht University Medical Center, P. Debyelaan 25, 6229 HX Maastricht, The Netherlands; 2 Department of Clinical Epidemiology and Medical Technology Assessment, Maastricht University Medical Center, P. Debyelaan 25, 6229 HX Maastricht, The Netherlands

**Keywords:** Lymphedema, LVA, Quality of life, Lymph-ICF, Indocyanine green, ICG

## Abstract

**Purpose:**

Lymphedema is a debilitating condition that significantly affects patient’s quality of life (QoL). The aim of this study was to assess the long-term outcomes after lymphaticovenous anastomosis (LVA) for extremity lymphedema.

**Methods:**

A single-center prospective study on upper and lower extremity lymphedema patients was performed. All LVA procedures were preceded by outpatient Indocyanine Green (ICG) lymphography. Quality of life measured by the Lymph-ICF was the primary outcome. Limb circumference, use of compression garments, and frequency of cellulitis episodes and manual lymphatic drainage (MLD) sessions were secondary outcomes.

**Results:**

One hundred consecutive patients, predominantly experiencing upper extremity lymphedema following breast cancer (*n* = 85), underwent a total of 132 LVAs. During a mean follow-up of 25 months, mean Lymph-ICF score significantly decreased from 43.9 preoperative to 30.6 postoperative, representing significant QoL improvement. Decrease in upper and lower limb circumference was observed in 52% of patients with a mean decrease of 6%. Overall mean circumference was not significantly different. Percentage of patients that could reduce compression garments in the upper and lower extremity group was 65% and 40%, respectively. Number of cellulitis episodes per year and MLD sessions per week showed a mean decrease of respectively 0.6 and 0.8 in the upper extremity and 0.4 and 1.0 in the lower extremity group.

**Conclusions:**

LVA resulted in significant QoL improvement in upper and lower extremity lymphedema patients. Limb circumference did not significantly improve but good results concerning compression garments, cellulitis episodes, and MLD sessions were obtained. Additionally, a simple and patient-friendly method for outpatient ICG lymphography is presented.

## Introduction

Lymphedema is a chronic, debilitating condition, characterized by abnormal accumulation of subcutaneous protein-rich fluid due to failure of the lymphatic drainage system [1–4]. It can affect any part of the body but is predominantly observed in the upper and lower extremities [5]. Lymphedema causes physical morbidity as it can lead to pain, skin tightness, heaviness, recurrent periods of cellulitis, and decreased range of motion [3–7]. Moreover, it affects psychological and emotional well-being instigating body image disturbances, anxiety, and depression [5, 8]. Consequently, lymphedema will significantly affect quality of life (QoL) and the ability to work and participate in social activities [8].

Treatment of lymphedema traditionally begins with complex decongestive therapy, consisting of a combination of skin care, exercise, compression therapy, and manual lymphatic drainage (MLD) [5, 9]. This treatment is time-consuming, and the effectiveness largely depends on the patient’s compliance [3]. Although this may result in enough symptomatic relief, none of the therapies will cure lymphedema, thus lifelong time-consuming therapy appointments and continuous use of compression garments are necessary, with a significant practical impact [2, 3, 8].

Therefore, over the last decades several surgical procedures have been proposed for the treatment of lymphedema, including lymphaticovenous anastomosis (LVA) [2, 10]. LVA is a minimal invasive method that redirects excessive lymph fluid from the oedematous limb into the venous system, by anastomosing lymphatic vessels to subdermal venules [1]. Although LVA surgery was already proven to be a valuable procedure in the 1970s [11, 12], it gained popularity after the introduction of supermicrosurgical techniques by Koshima et al. and the availability of indocyanine green (ICG) lymphography [13–16].

ICG lymphography is an innovative imaging technique that combines the administration of the fluorescent dye ICG with a near-infrared camera. This imaging modality enables direct visualization of the lymphatic system and is therefore used to determine the stage of lymphedema and evaluate the functionality of lymphatic vessels [10, 15, 17, 18]. In addition, ICG lymphography can guide surgeons during surgery by facilitating real-time decision-making, leading to more reliable and improved outcomes following LVA [1].

Over the years, numerous studies have investigated the efficacy of LVA as a treatment for lymphedema, demonstrating promising results [6, 9, 18–25]. Recent systematic reviews have demonstrated that limb circumference significantly decreased and QoL significantly increased following LVA surgery [2, 3]. However, most studies involved small sizes and reported short follow-up periods. In addition, a minority of previous studies have reported the effects of LVA on discontinuation of compression garments [9, 17, 22–24] and episodes of cellulitis [1, 4, 6, 26].

The aim of the current study was to assess the effect of LVA surgery in a large cohort of 100 lymphedema patients during a 24-month follow-up period. Special attention was paid to patients’ QoL, limb circumference, use of compression garments, the number of cellulitis episodes, and the number of MLD sessions.

## Methods

A single-institution prospective cohort study on 100 consecutive patients who underwent LVA procedures for primary and secondary lymphedema was performed at Maastricht University Medical Center between June 2015 and June 2018. Approval of the institutional review board was obtained (METC 2018-0869). Written informed consent was obtained from all included patients.

### Patient selection

Patients were eligible for LVA if they experienced subjective complaints of a confirmed unilateral upper or lower limb lymphedema, stage I to III according to the International Society of Lymphology (ISL) classification, and having undergone complex decongestive therapy for at least 3 months [27]. Additionally, patients were required to have patent lymphatic collecting vessels as visualized by preoperative ICG lymphography [15]. Exclusion criteria were as follows: active recurrent disease or metastasis in patients with history of malignancy, and the presence of an active skin infection. No limits were set on the time from the onset of lymphedema. Preoperative patient characteristics were obtained from the medical records of the included patients and from a prospectively maintained database. Variables obtained included the following: age, Body Mass Index (BMI), location (arm or leg), side and etiology of lymphedema, ISL stage, and ICG stage. The preoperative ISL and ICG staging were frequently, but not always, defined by the operating microsurgeon (SQ).

### ICG lymphography

**Fig. 1:**
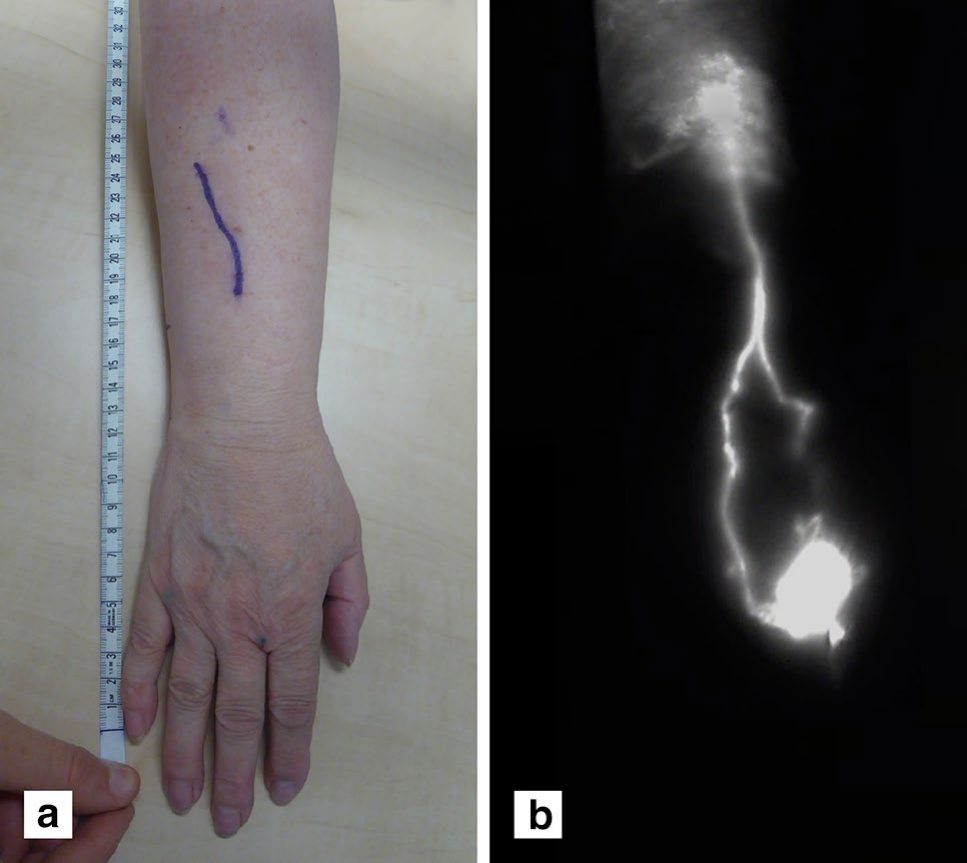
Outpatient ICG lymphography. Preoperative planning using ICG lymphography in the outpatient clinic (**a**) Lymph vessels visualized by ICG lymphography; linear pattern to stardust pattern and (**b**) corresponding markings for incision site, based on ICG lymphography

Although there is no substantial difference in the practical use of ICG lymphography, the time when to perform ICG lymphography is novel in this study. All patients underwent preoperative ICG lymphography during the outpatient appointment. A standard protocol was used for ICG lymphography: 0.02 ml (5 mg/ml) of ICG (PULSION® 25 mg for solution, PULSION Medical Systems SE, Feldkirchen, Germany) was subcutaneously injected in the second and fourth web spaces of the hand or foot. Patients were asked to wait in the waiting room for approximately 20 min, where they were able to drink a cup of coffee and complete the Lymph-ICF. Patients were regularly monitored by a nurse, despite the low incidence of side effects of ICG [28]. Meanwhile, the plastic surgeon was able to perform another consult before conducting the ICG lymphography. The fluorescence signal was mapped and recorded using a handheld near-infrared camera (Fluobeam®, Fluoptics, Grenoble France). Subsequently, the lymphatic vessels suitable for LVA surgery and the incision site for anastomosis were marked with a regular skin marker. These markings and relevant anatomical landmarks were captured in color pictures that were used during the operation to determine the place of incision (see Fig. 1).

To assess the severity of upper and lower extremity lymphedema, dermal backflow was categorized into six stages according to Narushima et al. Briefly, in stage 0, no dermal backflow pattern is seen. In stage I, a splash pattern is seen around the axilla or in the groin region. In stage II to IV, progressive stardust patterns are observed, and stage V represents a diffuse pattern in the whole limb [15].

### Surgical procedure

**Fig. 2:**
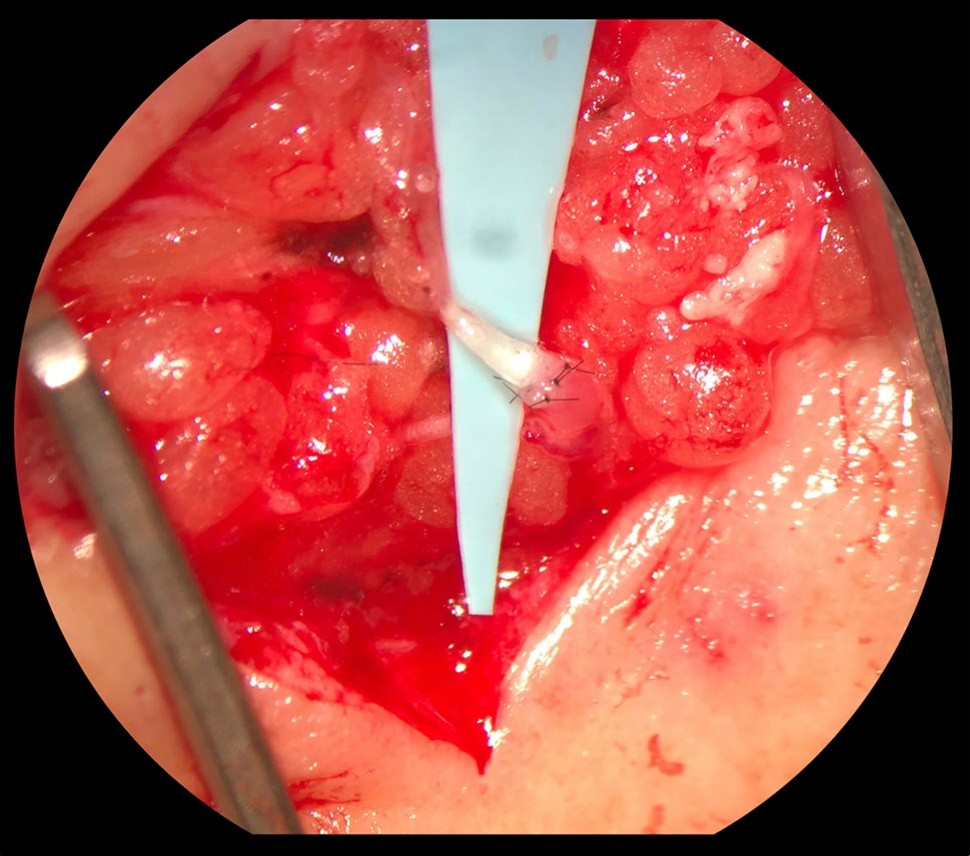
Intraoperative picture of an end-to-end lymphaticovenous anastomosis

The LVA procedures were performed under local anesthesia (bupivacaine hydrochloride 5 mg/ml with adrenaline 5 μg/ml) using the technique described by Koshima et al. [13]. All procedures were performed by one microsurgeon (SQ), within three to four months following the outpatient ICG lymphography. The incision was performed at the level of the lymphatic collecting vessel, as located using the aforementioned preoperative ICG lymphography. Using a microscope (ZEISS OPMI PENTERO 900;  × 25 to  × 50 magnification), one or more lymphaticovenous anastomoses were completed between a suitable lymphatic collecting vessel and a subcutaneous vein. In general, the anastomoses were performed in an end-to-end fashion using Ethilon 11-0. End-to-side anastomoses were created when the recipient vein was substantially larger than the lymphatic collecting vessel. Finally, the “milk test” was performed to evaluate anastomotic patency and evidenced lymphatic flow into the venules by gently stroking the lymph vessel. Hereafter, the skin was closed. All surgeries were performed within a maximum duration of 120 min. If not all potential lymphaticovenous anastomoses could be created within 120 min, a second or third procedure was planned. Figure 2 illustrates a completed LVA intra-operatively.

### Postoperative protocol

The postoperative protocol was followed as previously described [17]. In brief, patients were not allowed to wear compression garments or receive MLD in the first four weeks after surgery to minimize the chance of damaging the newly formed, fragile anastomosis. After this period, patients could choose, in consultation with the plastic surgeon and skin therapist, to restart compression garments and/or MLD sessions, depending on the presence of subjective complaints and the presence of swelling in the limb.

### Outcomes

Patients’ quality of life (QoL) was considered the primary outcome in this study. Secondary outcome measures included limb circumference, use of compression garment, annual episodes of cellulitis, and weekly MLD sessions. Patients’ QoL and circumference measurements were obtained preoperatively and postoperatively, presented in different follow-up periods: less than 2 months, 2 to 6 months, 6 to 12 months, 12 to 24 months, and > 24 months following LVA surgery. The final QoL or circumference measurement at the last outpatient appointment for each patient was also obtained. Similarly, the other outcomes (e.g., episodes of cellulitis) were evaluated during the last outpatient appointment per patient.

### Quality of life

Disease-specific QoL was measured by the Dutch Lymphedema Functioning, Disability, and Health questionnaire (Lymph-ICF) [29]. This is a validated questionnaire to evaluate limb specific symptoms in lymphedema patients using a Visual Analogue Score (VAS), with the advantage of a wide score range and high sensitivity [30, 31]. There are two versions: one for upper extremity and one for lower extremity lymphedema. Both comprise five domains: physical function, mental function, household activities, mobility activities, and life and social activities. The scores range from 1 to 100: a lower score on the questionnaire represents a better QoL. A decrease in VAS of more than 10 in the total score was considered to be statistically significant (*p* < 0.05).[29].

### Limb circumference

Circumference change in the operated arm and leg were calculated with the Upper and Lower Extremity Lymphedema index (UEL- and LEL-index) [32]. Limb circumference was measured at standardized landmarks on the arm or leg, and together with the patient’s BMI, the UEL- or LEL-index was calculated [32]. Patients had to remove their compression garment 24 h prior to the follow-up moment in order to achieve a more reliable measurement [17].

### Statistical analysis

Continuous variables were reported as mean with standard deviation. Categorical data were reported as frequency and proportion. To examine the effect of LVA, differences in preoperative and postoperative means were analyzed using the Paired Samples T-Test. Differences in proportions were analyzed using the Chi-squared test or McNemar test for independent and dependent proportions, respectively. Differences in change scores (preoperative minus postoperative) between the operated limb and non-operated limb were tested using linear regression analyses. To deal with loss to follow-up, postoperative outcome measurements were compared to the related preoperative outcome measurements for the specific number of patients.

The relationship between the number of LVAs, ICG stage (0–3 vs. 4–5), circumference difference, follow-up months, compression garment (no versus yes), and success on QoL was computed using linear regression analysis and quantified as unstandardized beta (B) with a 95% confidence interval (CI).

Results were analyzed using IBM SPSS Statistics for Windows, version 24 (IBM corp. ®, Armonk, N.Y, USA). A *p*-value < 0.05 was considered to be statistically significant.

## Results

### Demographics

One hundred consecutive patients with a mean age of 57.1 years underwent a total of 132 operations, in which 270 anastomoses were completed. Since some patients had more potential LVAs than could be performed in 120 min, the total number of LVAs was split over two or three procedures. The majority of patients (*n* = 70) underwent a single operation with a mean number of 2.7 LVAs. Twenty-eight patients underwent 2 operations with a mean number of 3.93 LVAs and only 2 patients underwent 3 operations with a mean number of 7.5 LVAs. Mean follow-up was 25.0 months. LVAs were predominantly performed in women with unilateral upper limb lymphedema following breast cancer treatment (*n* = 85), classified as ISL stage IIA or ICG stage 3. Table 1 summarizes the main characteristics of the patients included in the current study.

**Table 1:** Demographics and clinical information

	Mean, SD	*N*
Patients		100
Operations		132
LVAs		270
Gender		
Female		94
Male		6
Age (years)	57.1 ± 10.6	
BMI (kg/m^2^)	26.3 ± 4.9	
Location of lymphedema		
Arm		85
Leg		15
Etiology of lymphedema		
Primary		6
Secondary		94
Affected side		
Left		46
Right		54
ISL stage^a^		
I		4
IIA		69
IIB		25
III		2
ICG stage^b^		
1		1
2		19
3		45
4		26
5		9
Follow-up (months)	25.0 ± 10.9	
Number of operations per patient	1.3 ± 0.5	
1 operation		70
2 operations		28
3 operations		2
Number of LVAs per patient	2.7 ± 1.4	
1 LVA		16
2 LVAs		39
3 LVAs		21
4 LVAs		15
5 LVAs		5
6 LVAs		1
7 LVAs		2
8 LVAs		1

^a^International Society of Lymphology

^b^Stage according to ICG lymphography

### Quality of life

**Fig. 3:**
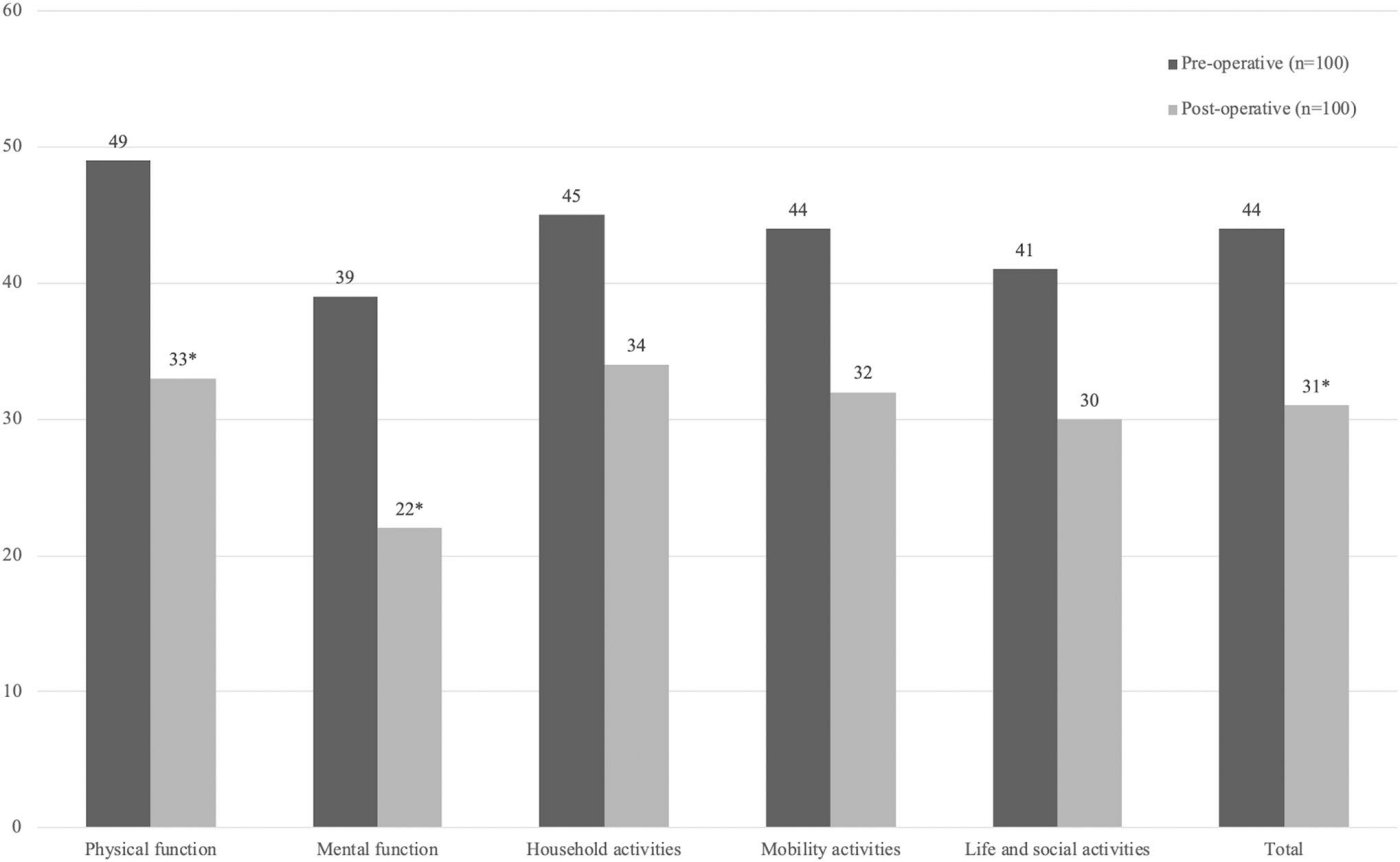
Mean Lymph-ICF preoperatively and postoperatively. Final Lymph-ICF measurement at last outpatient appointment for each patient (*n* = 100) over a mean follow-up 24.5 ± 10.9 months. Analysis: Paired Samples *T*-Test. *Statistically significant difference: A decrease of 10, 15, 12, 23, 15, and 14 in total score, physical function, mental function, household activities, mobility activities, and life and social activities were considered a statistically significant difference (*p* < 0.05) [29]

After a mean follow-up of 25 months, the mean total lymph-ICF score showed a decrease of 13.3 (*p* < 0.001); 43.9 ± 19.0 preoperative to 30.6 ± 20.2 postoperative (*n* = 100). See Fig. 3. This decrease was independent of duration of follow-up. The response rate in the different follow-up periods ranged from 53 to 67%. Of all 100 consecutive patients, only 56 patients had an end-point available at 24 months follow-up as not all patients returned to the outpatient appointment in each follow-up period and the questionnaire was not always filled in completely. See Tables 2 and 3.

**Table 2:** Preoperative versus postoperative total Lymph-ICF score for upper and lower extremities

Follow-up (FU) period	FU (months) Mean ± SD	Lymph-ICF score^a^			
		Preoperative Mean ± SD	Postoperative^b^ Mean ± SD	Mean difference	*P-value*
< 2 months (n = 67)^c^	1.3 ± 0.5	46.8 ± 17.6	27.3 ± 18.1	− 19.5	< *0.0001*
2–6 months (*n* = 56)^c^	3.7 ± 1.0	43.0 ± 18.4	29.5 ± 19.6	− 13.5	< *0.0001*
6–12 months (*n* = 53)^c^	8.0 ± 1.9	43.0 ± 18.8	26.4 ± 18.0	− 16.6	< *0.0001*
12–24 months (*n* = 59)^c^	16.0 ± 3.6	45.3 ± 16.6	31.3 ± 18.6	− 14.0	< *0.0001*
> 24 months (*n* = 56)^c^	32.1 ± 6.4	39.6 ± 19.4	27.5 ± 20.6	− 12.1	< *0.0001*

^a^Calculated using the Paired Samples T-Test

^b^Mean difference in total Lymph-IC score between preoperative and postoperative score for the reported number of patients

^c^The number of patients included in the analysis

**Table 3:** Linear regression analysis with Lymph-ICF difference as dependent variable

Independent variable	Lymph-ICF score difference^a^		
	*B*^b^	95% CI	*P-value*
Number of LVAs	− 2.89	− 5.29 to − 0.50	*0.018*
ICG stage (1–3 vs 4–5)	− 3.22	− 10.20 to 3.75	*0.361*
Circumference difference	− 0.09	− 0.34 to 0.15	*0.448*
Follow-up (months)	0.03	− 0.28 to 0.34	*0.852*
Compression garment (no vs yes)^c^	− 5.24	− 11.98 to 1.50	*0.126*

^a^Lymph-ICF score difference is calculated by subtracting the post-OR lymph-ICF score from the pre-OR lymph-ICF score

^b^Unstandardized beta (B):Calculated using linear regression analysis. A negative value means a decrease in Lymph-ICF, representing an increase in Quality of Life

^c^No: discontinuation of compression garment, Yes: partial discontinuation and continuation of compression garment

When concerning the final Lymph-ICF measurement at the last outpatient appointment for each patient (*n* = 100), a lower postoperative total score in each domain of the Lymph-ICF was observed. However, only in domain ‘physical function’ and ‘mental function’ a decrease of more than 10 was observed, representing a statistically significant decrease (*p* < 0.05) [29]. See Fig. 3.

Following LVA surgery, a lower Lymph-ICF score was observed in 84% of patients, with a mean decrease of 17.7 ± 14.0. A decrease of more than 10 in Lymph-ICF score was observed in 51% of patients, with a mean decrease of 25.8 ± 12.1 (*p* < 0.05). No relationship between Lymph-ICF score and preoperative ICG stage, difference in limb circumference, follow-up period, and the need to use compression garment was found. However, the number of LVAs was related to a decrease in Lymph-ICF, representing a better QoL (B − 2.89, 95% CI − 5.29 to − 0.50, *p* = 0.018). See Table 3.

### Limb circumference

A decrease in circumference was observed in 52.1% of all patients (*n* = 50). When analyzed separately, a decrease in UEL-and LEL-index was observed in 53% (*n* = 43) and 46.7% (*n* = 7) of patients, respectively, with a mean decrease of both UEL- and LEL-index of 6%.

The mean difference in UEL-index during the last outpatient appointment per patient (*n* = 81) was + 0.5 (*p* = 0.686): 122.9 ± 19.9 preoperative to 123.4 ± 22.3 postoperative. Four patients were excluded from circumference analyses, since they were wearing compression garments during follow-up moments. The mean difference in UEL-index of the operated arm and the non-operated arm was + 0.5 and − 0.4, respectively (*p* = 0.420). The mean differences in preoperative and postoperative UEL-indices over the different follow-up periods are presented in Table 4.

**Table 4:** Preoperative versus postoperative upper extremity lymphedema (UEL) indices (*n* = 85)

FU period	FU (months) Mean ± SD	Circumference (cm)^a^			
		Preoperative circumference Mean ± SD	Postoperative^b^ Circumference Mean ± SD	Difference Mean ± SD	*P-value*
< 2 months (*n* = 73)^b^	1.34 ± 0.5	122.1 ± 20.5	122.4 ± 22.2	+ 0.3 ± 10.7	*0.760*
2–6 months (*n* = 52)^b^	3.6 ± 0.9	124.4 ± 20.6	123.2 ± 21.7	− 1.2 ± 8.8	*0.334*
6–12 months (*n* = 39)^b^	8.1 ± 2.0	121.4 ± 17.7	123.4 ± 19.8	+ 2.0 ± 9.7	*0.207*
12–24 months (*n* = 40)^b^	14.4 ± 3.0	122.1 ± 17.5	121.7 ± 21.1	− 0.4 ± 8.7	*0.787*
> 24 months (*n* = 18)^b^	27.5 ± 4.3	119.8 ± 13.8	116.7 ± 15.0	− 3.1 ± 8.7	*0.144*

^a^Calculated using the Paired Samples *T*-Test

^b^Mean difference in UEL-index preoperative and postoperative score for the reported number of patients

^c^The number of patients included in the analyses

The mean difference in LEL-index during the last outpatient appointment per patient (n = 15) was + 2.3 (*p* = 701): 265.9 ± 54.2 preoperative to 268.2 ± 56.7 postoperative. The mean difference in LEL-index of the operated leg and the non-operated leg was + 2.3 and + 7.3, respectively (*p* = 0.961).

### Other outcomes

The majority of patients with upper and lower extremity lymphedema experienced a positive effect of the LVA procedure. Overall, 43% of all patients completely discontinued the use of compression garments at the last outpatient appointment. Eighteen percent of all patients reported the use of compression garments only during some activities (e.g., sports, gardening). The continuation rate was 35.5% in patients with upper extremity lymphedema, in contrast to 60% in patients with lower extremity lymphedema.

The proportion of patients experiencing episodes of cellulitis was lower in both groups (*p* < 0.01). Although the mean decrease in number of cellulitis episodes was lower in patients with upper (− 0.6) and lower extremity lymphedema (− 0.8), the mean difference in the lower extremity group was not found to be statistically significant (*p* = 0.492) . This was probably due to the small sample size (*n* = 15).

A mean decrease in MLD sessions per week in patients with upper (− 0.4) and lower extremity lymphedema (− 1.0) was observed (*p* < 0.01). See Table 5 for differences between the arm and leg group.

**Table 5:** Compression garments and patient-reported outcomes

	Total	*P-value*	Arm	*P-value*	Leg	*P-value*
Compression garments (*n*)	*100*		*85*		*15*	
Discontinuation (%)	43		47.1		20	
Partial discontinuation (%)	18		17.6		20	
Continuation (%)	39	*NE*	35.3	*NE*	60	*NE*
Positive effect^b^ (*n*)^*a*^	*100*		*85*		*15*	
Yes (%)	80		77.6		93.3	
No (%)	20	< *0.001*	22.4	< *0.001*	7.6	*0.005*
Patients experiencing cellulitis^a^ (*n*)^*c*^	*98*		*83*		*15*	
Before operation (%)	38.8		41		26.7	
After operation (%)	23.5	*0.001*	26.5	*0.007*	6.7	*0.031*
Cellulitis episodes per year^a^ (*n*)^*d*^	*98*		*83*		*15*	
Before operation (Mean ± SD)	1.1 ± 1.9		1.0 ± 1.6		1.4 ± 3.3	
After operation (Mean ± SD)	0.5 ± 1.3	*0.006*	0.4 ± 1.0	*0.001*	0.6 ± 2.3	*0.492*
MLD sessions per week^a^ (*n*)^*d*^	*82*		*70*		*12*	
Before operation (Mean ± SD)	1.3 ± 1.0		1.2 ± 0.8		1.9 ± 19	
After operation (Mean ± SD)	0.8 ± 0.8	< *0.001*	0.8 ± 0.71	< *0.001*	0.9 ± 1.4	*0.005*

^a^Calculated using the Chi-Square test

^b^Outcome reported by patient

^c^Calculated using the McNemar test

^d^Calculated using the Paired Samples *T*-Test

## Discussion

This prospective cohort study comprising 100 upper and lower extremity lymphedema patients showed significant QoL improvement after LVA surgery. Two-thirds of extremity lymphedema patients were able to reduce (i.e., partially or completely discontinue) the use of compression garments. Moreover, the postoperative number of cellulitis episodes and MLD sessions decreased for both types of lymphedema. Additionally, a simple and patient-friendly method for outpatient ICG lymphography is presented.

Significant mean QoL improvements after LVA surgery were reported with consistent results in all follow-up periods until mean follow-up of 32 months. A recent systematic review on QoL following surgical treatment of lymphedema revealed that the majority of previous studies report QoL improvement solely based on the patient’s feelings [7]. The reported proportions (range 57–100%) are consistent with the 80% of patients experiencing a positive effect in the current study [7]. The other studies did use validated tools to asses QoL. However, these are studies with small sample sizes (range 10–74 patients) and relatively short follow-up (range 6–12 months) [10, 22, 23, 33–35]. To the best of our knowledge, the current study may cover the largest population with the longest follow-up, assessing QoL improvement over different periods of time. Moreover, the Lymph-ICF was used, which has recently been assessed as one of the most complete and accurate questionnaires to assess QoL in lymphedema patients [30]. Improvement in QoL was related to the total number of LVAs performed per patient in this study. This could be explained by previous observation that a higher of anastomoses could be associated with a better patency rate and the suggestion of a positive correlation between a patent anastomosis and clinical improvement [17].

The overall mean limb circumference did not improve. This result is consistent with previous findings [17, 22]. In the current study, a decrease in limb circumference in terms of UEL- and LEL-index was observed in half the patients, with a mean decrease of 6%. The difference with a previous systematic review, reporting a weight mean circumference reduction of 8.5%, could be due to the high heterogeneity of patient population and assessment modalities in previous studies [3]. Although one may conclude that LVA treatment was not effective when minimal decrease in circumference is observed, lymphedema progression may be ceased due to the procedure [10].

Only 35% of upper extremity lymphedema patients needed to continue their compression garments after LVA, compared to 60% of lower extremity lymphedema patients. These results are in line with previous studies reporting continuation rates (range 15–66%) [9, 17, 22, 23, 36]. Since a majority of these studies report a maximum follow-up of 12 months, more research similar to the current study is recommended to confirm the long-term effects of LVA surgery on compression garment usage. The difference between continuation rates for upper and lower extremity lymphedema patients supports the finding by Chang et al., who concluded that LVA in the lower extremity was not as effective compared to the upper extremity [18].

A reduction of more than 50 percent in mean cellulitis episodes was observed for both upper and lower extremity lymphedema cases. This is an important finding, since 23–35% of lymphedema patients experience recurrent and progressive cellulitis, and it has a tremendous impact in their quality of life.[5, 6, 37]. Cellulitis leads to a vicious cycle of lymphatic vessel destruction, lymphedema, and recurrent cellulitis episodes. Few studies have shown that LVA can interrupt this cycle and reduce the number of cellulitis episodes [1, 4, 6, 26]. The current study underlines these findings, with the positive observation that this is also the case for the longer term.

Regarding the need for MLD, 20% of patients were able to cease MDL sessions, while the remaining patients continued MLD to a lesser degree (51%) or at the same frequency (29%), resulting in a significantly lower mean number of MLD sessions for all patients. Although previous studies concerning MLD as an outcome measurement are scarce, these results are in line with previous research and confirm a consistent longer-term result [17].

In this study, a simple and patient-friendly method for outpatient ICG lymphography is presented. Previously, intraoperative mapping of lymphatics was reported with the subsequent disadvantage of lengthening duration of the operation [18, 19, 21, 23, 38, 39]. Since all ICG injections were well tolerated, patients experienced the method as patient-friendly, knowing that these would not be different in the operation room. Moreover, patients felt safe, as they were observed for side effects. Additionally, we believe that the presented method can save time in the operation room, since all lymphatic vessels were identified exactly at the incision site that was marked and photographed in outpatient clinic making intraoperative ICG lymphography redundant. However, the exact time saving effect remains to be investigated.

In the current study, clinical heterogeneity was low as predominantly female patients with secondary upper limb lymphedema with a small standard deviation for age and BMI were included. However, three-quarters and two-thirds of the included patients presented with early ISL stages and low ICG stages, respectively. These factors could have been a beneficial factor since LVA might have been less effective in patients with advanced lymphedema and patients presenting with ICG stage IV or more [17]. Nevertheless, no relationship between ICG stages 1 to 3 and QoL improvement was observed in this study. Although not assessed in the current study, the use of an experienced microsurgeon who operated all patients using the same surgical technique may have affect the outcome. Furthermore, the postoperative treatment protocol in the current study differs from studies in which patients directly start wearing compression garments following surgery [23]. However, there is currently no evidence on the optimal conservative treatment protocol following LVA surgery [39].

The limitations that are worthy to mention in the current study are the following: the number of patients included in each individual follow-up moment is limited. Patients were unfortunately lost to follow-up for as patients found it not useful to return to the outpatient clinic a long time after the operation and patients needed to travel a long distance as patients from all over the country visit our institution for lymphedema treatment. Nonetheless, this is still the largest prospective cohort study evaluating multiple relevant outcomes following LVA procedure. In current study, no correlation between patency and QoL improvement after LVA was explored. However, previous work by our group on 25 patients, who were also included in current study, showed that 76% of patients had at least one patent anastomosis after 12 months, and a positive correlation between a patent anastomosis and clinical improvement was observed [17]. Furthermore, due to the maximum operation duration of 120 min, one-third of patients required a second or third operation to perform all potential LVAs. This may entail financial burdens for patients, since LVA surgery is not reimbursed by every health care insurance companies over the world.

Another remark, which also applies to previous studies, is that the possible effect of arm dominance on patients’ QoL was not taken into account. Notwithstanding the promising results, randomized controlled studies are required to provide higher evidence for the effectiveness of LVA surgery [40].

## Conclusion

LVA resulted in significant QoL improvement of upper and lower extremity lymphedema patients. Limb circumference did not significantly improve, but good results concerning discontinuation of compression garments (especially for the upper extremity lymphedema group), decrease in cellulitis episodes, and MLD sessions were observed. Additionally, a simple and patient-friendly method for outpatient ICG lymphography is presented which facilitates preoperative decision-making.

## Data Availability

The datasets generated during and/or analyzed during the current study are available from the corresponding author on reasonable request.

## Abbreviations

BMI: Body mass index
CI: Confidence interval
ICG: Indocyanine green
ISL: International society of lymphology
LEL-index: Lower extremity lymphedema index
LVA: Lymphaticovenous anastomosis
Lymph-ICF: Lymphedema functioning, disability and health questionnaire
MLD: Manual lymphatic drainage
OR: Odds ratio
QoL: Quality of life
UEL-index: Upper extremity lymphedema index
VAS: Visual analogue score
